# Synergistic inhibitory effects of deferasirox in combination with decitabine on leukemia cell lines SKM-1, THP-1, and K-562

**DOI:** 10.18632/oncotarget.16583

**Published:** 2017-03-27

**Authors:** Nianyi Li, Qinfen Chen, Jingwen Gu, Shuang Li, Guangjie Zhao, Wei Wang, Zhicheng Wang, Xiaoqin Wang

**Affiliations:** ^1^ Department of Haematology, Huashan Hospital, Fudan University, Shanghai, China

**Keywords:** synergistic effect, deferasirox, decitabine, iron chelation therapy, demethylation

## Abstract

A multi-center study from the French Myelodysplastic Syndrome (MDS) Group confirmed that iron chelation therapy is an independent prognostic factor that can increase the survival rate of patients who are suffering from transfusion-dependent low-risk MDS. In this study, we aimed to explore this clinical phenomena *in vitro*, by exploring the synergistic effect of the iron chelator Deferasirox (DFX) and the DNA methyl transferase inhibitor Decitabine (DAC) in the leukemia cell lines SKM-1, THP-1, and K-562. Treatment with both DFX or DAC promoted apoptosis, induced cell cycle arrest, and inhibited proliferation in all three of these cell lines. The combination of DFX and DAC was much greater than the effect of using either drug alone. DFX showed a synergistic effect with DAC on cell apoptosis in all three cell lines and on cell cycle arrest at the G0/G1 phase in K-562 cells. DFX decreased the ROS levels to varying degrees. In contrast, DAC increased ROS levels and an increase in ROS was also noted when the two drugs were used in combination. Treatment of cells with DAC induced re-expression of ABAT, APAF-1, FADD, HJV, and SMPD3, presumably through demethylation. However the combination of DAC and DFX just had strong synergistic effect on the re-expression of HJV.

## INTRODUCTION

Myelodysplastic syndrome (MDS) is a malignant condition of bone marrow stem cells. It is characterized by ineffective hemopoiesis of stem or progenitor cells, which leads to peripheral blood cytopenias and may progress to acute myeloid leukemia in some patients [1]. The International Prognostic Scoring System (IPSS) groups MDS patients into four prognostic categories and the treatment of MDS varies according to risk group [2]. The clinical outcome for MDS remains discouraging, although treatments such as immunomodulatory agents, low-dose chemotherapy, and allogeneic blood or marrow cell transplantation have been used, and “Active therapy” was given only when the disease progressed to AML, or resembled AML, in terms of severe cytopenias [3].

Currently, the most frequent treatment given to patients with MDS is supportive care. This has led a substantial subgroup of MDS patients to eventually develop transfusion dependency, resulting in secondary iron overload in which elevation of serum ferritin (SF), heart failure, and liver dysfunction are often observed [4]. Since the body has no physiological mechanism to excrete excess iron, in order to avoid secondary iron overload, patients receiving long-term blood transfusion should be treated with iron chelation therapy (ICT) according to the MDS Foundation's guidelines [5].

In a recent multicenter study, the French Myelodysplastic Syndromes Group (GFM) confirmed that iron chelation therapy appears to improve survival in heavily transfused, lower-risk MDS [2]. In this study, they analyzed survival and cause of death in ninety-seven low, or intermediate-1 (int-1) -risk patients from eighteen centers, regularly transfused as outpatients, chelated or not, who were studied for a month and followed for 2.5 years. In this heavily transfused cohort of low- and int-1-risk MDS patients, a significant survival advantage was seen in chelated patients, particularly in those who had received sufficiently intensive chelation therapy [2]. However, there is no precise understanding of the reason for this survival advantage provided by ICT.

However, anecdotally, it is believed that the reason iron therapy improves a patient's prognosis is due to an overall reduction in the iron load on the heart, liver, brain, lung, kidney, and other vital organs. However, numerous other studies have shown that some iron chelation drugs have an anti-cancer action including Deferasirox. Deferasirox (DFX) is a new oral iron chelator approved by the US Food and Drug Administration (FDA) and the European Medicines Agency (EMA) for clinical use since 2010. DFX shows better safety, tolerability, convenience, with fewer adverse effects compared to Deferoxamine (DFO), which had been the treatment of choice for iron overload for the last 40 years [6]. Breccica *et al*. summarized the potential pathogenic mechanisms responsible for hematologic improvement induced by DFX [7], such as a direct effect on neoplastic clone or the bone marrow environment, promoting iron release from iron stores, allowing use by hemopoietic tissue, reduction of the levels of reactive oxygen species (ROS)[8], inhibition of m-TOR signaling [9], and inhibition of NF-kB signaling [10].

The DNA methyl transferase inhibitors, 5-azacytidine and 5-aza-2′deoxycytidine (Decitabine (DAC)) were approved as chemotherapeutics for the treatment of MDS in 2004 and 2006, respectively. These drugs both improve the outcome for patients with high risk MDS, and ameliorate the cytopenias of MDS and decrease the percentage of blasts in the bone marrow [11]. Numerous clinical trials have shown DAC to be effective in MDS. In some iron chelation studies, including one conducted by GFM, some of the patients were also taking DAC. As a DNA methyl transferase inhibitor, the DAC mechanism of action involves relaxation of chromatin structure by causing DNA demethylation or hemi-demethylation. Chromatin relaxation leads to an increase in transcription factor binding to promoters thereby increasing gene transcription of target genes.

Based on these considerations, we hypothesized that the iron chelator DFX, in addition to improving median overall survival (OS) in MDS and AML patients, may possess a distinct anti-tumor activity that also leads to an improvement in prognosis for MDS and AML patients. Furthermore, Pogribny *et al*. have provided experimental proof for an interdependence between iron and epigenetic regulatory mechanisms and suggested that modification of intracellular iron metabolism by itself may enhance the efficacy of epigenetic therapy in breast cancer [12]. Hence, in this study we tried to understand if a similar relationship existed in MDS and AML.

The aim of the present work was to study the effect of DFX and DAC, and combinations of both, on cell viability, apoptosis, and cell-cycle progression in the human leukemia cell lines SKM-1, THP-1, and K-562 *in vitro*. We also examined ROS levels in each of these cell types, since ROS play a critical role in hematopoietic stem cell (HSC) metabolism [8]. In our previous work, we discovered five MDS-related genes that are simultaneously hyper-methylated and transcriptionally downregulated [13–15]. Therefore, in this study, we also examined the expression of these five genes, before and after treatment with DFX and DAC, in the hope of finding if ICT can promote their re-expression.

## RESULTS

### Combination treatment with DFX and DAC showed a greater anti-proliferative effect on leukemia cell lines compared to single-drug treatment

**Figure 1:**
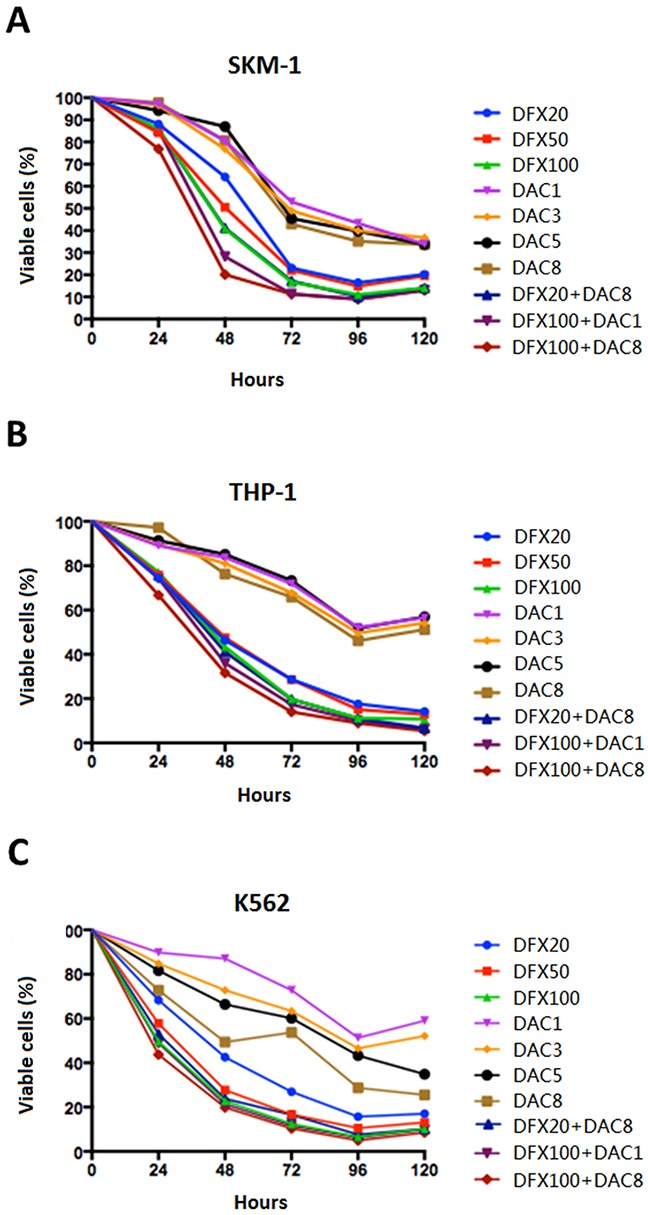
Effect of DFX and DAC on the viability of three leukemia cell lines (SKM-1, THP-1, and K562) both alone and in combination Cell viability was determined using the CCK-8 assay and the differences in cell growth following exposure to DFX and DAC, both alone and in combination, was determined. The data are shown as mean±S.D. values from three independent experiments.

To evaluate anti-proliferative effects, the three leukemia cell lines, SKM-1, THP-1, and K-562 cells were treated with DAC, DFX, or a combination of DAC and DFX for 24, 48, 72, 96, and 120 h. The results revealed that increasing concentrations of both drugs, ranging from 20 to 100 μM of DFX and 1 to 8 μM of DAC, all significantly suppressed cell line viability in dose- and time-dependent manners. Based on these single dose response studies, the most effective concentration and minimum effective dose of each drug was picked to create different drug combinations including 20 μM DFX with 8 μM DAC, 100 μM DFX with 1 μM DAC, and 100 μM DFX with 8 μM DAC, in order to evaluate the effect of DFX in combination with DAC. Data with DFX and DAC in different combinations suggested predominantly synergistic or additive interactions for proliferation in all cell lines. In SKM-1 cells, the cell viabilities when treated with 100 μM DFX or 8 μM DAC alone for 24, 48, 72, or 96 h were 86.01%, 40.75%, 16.68%, and 11.04% and 97.78%, 80.68%, 42.94%, and 35.15%, respectively. The combination of the two drugs (100 μM DFX plus 8 μM DAC) for 24, 48, 72, or 96 h decreased cell viability to 76.85%, 20.16%, 11.32%, 8.90%, respectively. Other doses also showed the same trend (Figure 1A). These results suggested that the combined treatment of DFX and DAC showed a greater anti-proliferative effect on the SKM-1 cell line, compared to treatment using DFX or DAC alone. This synergistic anti-proliferative effect of DFX and DAC was also observed in THP-1 and K-562 cells (Figure 1B, 1C).

### The combination of DFX and DAC induced more cells to undergo apoptosis than single-drug treatment

**Figure 2:**
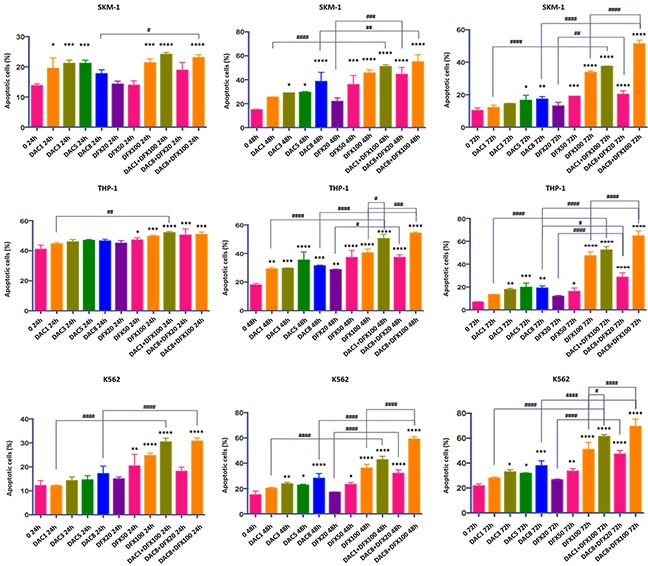
Induction of apoptosis by annexin V staining in SKM-1, THP-1, and K562 cells after 24 h, 48 h, and 72 h treatment with DFX and/or DAC Each bar represents the mean ± SD.

**Figure 3:**
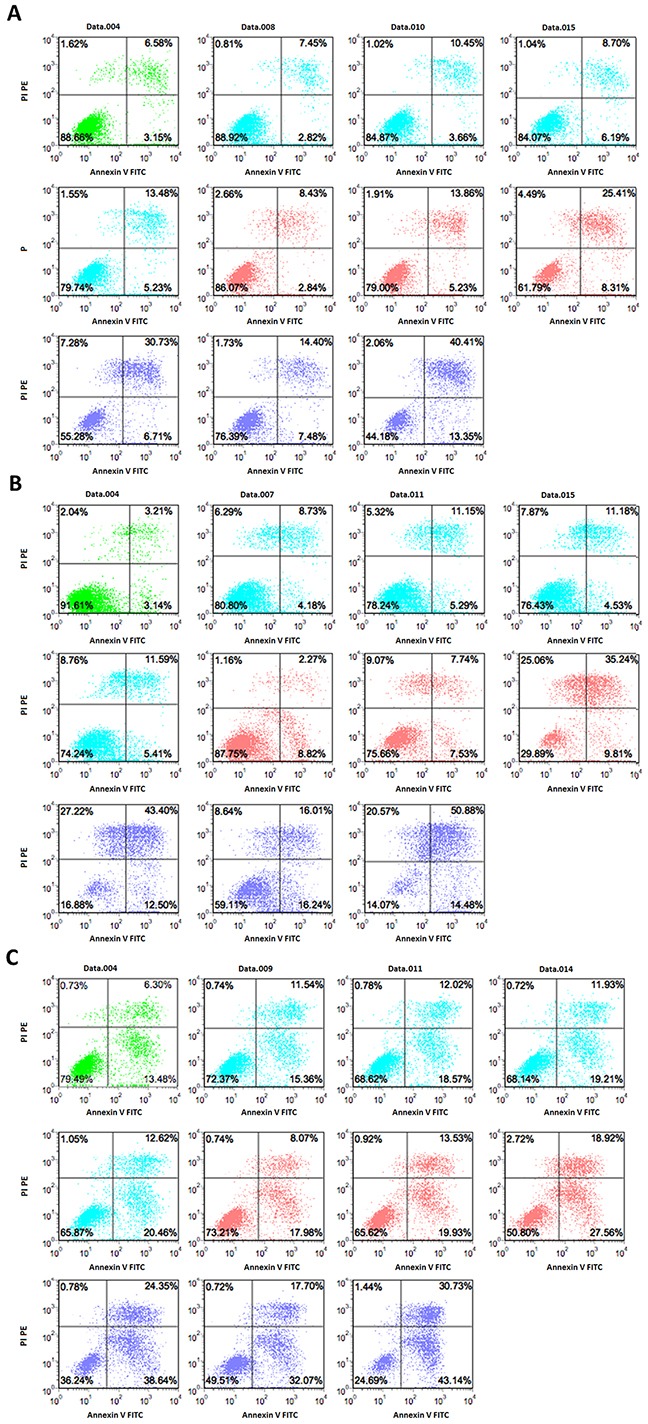
Induction of apoptosis by DFX and/or DAC determined by double staining with Annexin V–FITC and PI SKM-1, THP-1, and K562 cells were incubated with different concentrations of drugs for 72 h. The flow cytometry profile represents Annexin V–FITC staining on the x-axis and PI on the y-axis. Representative data from two different experiments are shown. Control is represented in green. The DAC group is represented in blue at the following concentrations, 1 μM, 3 μM, 5 μM, and 8 μM. The DFX group is represented in red at the following concentrations, 20 μM, 50 μM, and 100 μM. The DAC and DFX groups are shown in purple at the following concentrations, 1 μM DAC with 100 μM DFX, 8 μM DAC with 20 μM DFX, and 8 μM DAC with 100 μM DFX.

Cell apoptosis is generally used as an indicator for growth inhibition of cells following drug treatment. In order to determine if cell apoptosis was involved in growth inhibition in these cell lines induced by either individual or combined treatment of DFX and DAC, we performed flow cytometry analysis using Annexin V/PI staining. The apoptotic fraction was considered as both early apoptotic cells and late apoptotic cells. As shown in Figures 2 and 3, both drugs alone showed a significant dose- and time-dependent induction of apoptosis in all three cell lines. In SKM-1 cells, the fraction of apoptotic cells was 33.74±0.96% and 17.26±1.65% after 72 h treatment with 100 μM of DFX or 8 μM of DAC, respectively. The fraction of apoptotic cells increased to 51.3±2.28% with the combination of both drugs (P<0.0001). In THP-1 cells, the apoptotic fraction was 47.32±3.56% following treatment with 100 μM DFX alone for 72 h, and 19.09±1.91% following treatment with 8 μM DAC for 72 h alone. This fraction increased to 64.72±4.39% with the combination treatment (P<0.0001). In K562 cells, the apoptotic fraction was 50.85±5.87% following 100 μM DFX treatment for 72 h, and 37.75±4.06% following treatment with 8 μM DAC treated for 72 h. This fraction increased to 69.34±5.95% with combination treatment (P<0.0001). These results suggested that the combined use of these two drugs induced cell apoptosis more efficiently than either drug alone.

### Effect of treatment with DFX and DAC alone or in combination on cell-cycle distribution

**Figure 4:**
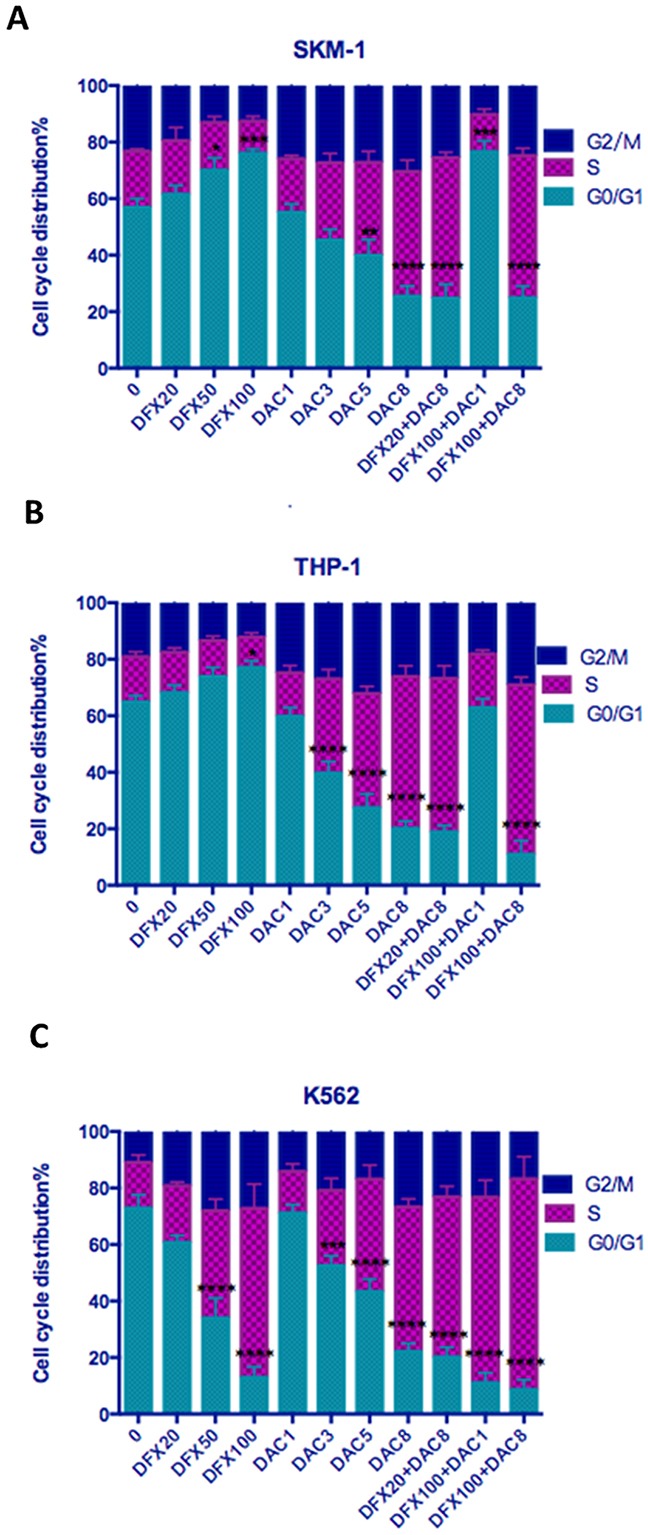
Effect of DFX and/or DAC on cell cycle progression in leukemia cell lines SKM-1, THP-1, and K562 cells were treated with drugs for 48 h and their cell-cycle distribution was evaluated using flow cytometry. Each bar represents the percentage of cells in G0/G1, S, and G2/M phases presented as mean ± SD value.

To examine whether the growth inhibitory effect of these compounds could be explained by alterations in the cell-cycle, we examined the effects of DFX and DAC alone, and in combination, on the cell cycle of these three leukemia cell lines. We found that DFX caused SKM-1 and THP-1 cells to arrest in the G0/G1 phase in a dose-dependent manner. However, in K562 cells, treatment with DFX arrested cells at S phase. When treated with DAC, all three cell lines showed a dose-dependent arrest in S phase. Interestingly, when cells were treated with a combination of the two drugs at various concentrations, the actual concentrations of the two drugs used in each combination seemed to determine which phase of the cell cycle the cells arrest in. For example, the percentage of cells in the G0/G1 phase in untreated SKM-1 cells was 56.84±3.14%. When treated with 20 μM DFX or 100 μM DFX for 48 h, the percentages of SKM-1 cells in the G0/G1 phase were 61.47±3.16% and 76.00±1.51%, respectively. When SKM-1 cells were treated with 1 μM DAC or 8 μM DAC for 48 h, the percentages of cells in the G0/G1 phase were 55.06±3.03% and 25.49±3.60%, respectively. After co-treatment with 8 μM DAC or DFX at 20 μM or 100 μM, the percentages of cells in the G0/G1 phase decreased to 24.74±4.97% and 24.87±4.17%. This trend was similar for treatment with DAC. After treatment with 1 μM DAC combined with 100 μM DFX, the percentage of cells in the G0/G1 phase increased to 76.61±3.82% (P<0.01). This trend was similar for treatment with DFX. In THP-1 cells, we obtained similar results; when a lower dose of DFX was combined with a larger dose of DAC, the percentage of cells in the G0/G1 phase decreased. Conversely, when a larger dose of DFX was combined with a lower dose of DAC, the percentage of cells in the G0/G1 phase increased. In K562 cells, both drugs decreased the percentage of cells in the G0/G1 phase and, when treated in combination, the additive effect was very evident. In untreated K-562 cells, the percentage of cells in the G0/G1 phase was 72.89±4.72%. When treated with either 100 μM DFX or 8 μM DAC for 48 h, the percentages of cells in the G0/G1 phase were 12.95±3.80% and 22.13±2.88%, respectively. After co-treatment with 100 μM DFX and 8 μM DAC, the percentage of cells in the G0/G1 phase decreased to 8.66±3.48% (P<0.01). (Figure 4 and Table 1).

**Table 1:** Cell cycle distribution after treatment with DFX and/or DAC for 48h

	control	DFX 20uM	DFX 50uM	DFX 100uM	DAC 1uM	DAC 3uM	DAC 5uM	DAC 8uM	DFX20uM +DAC8uM	DFX100uM +DAC1uM	DFX100uM +DAC8uM
**SKM-1**	56.84 ±3.14	61.47 ±3.16	70.03 ±4.35	76.00 ±1.51	55.06 ±3.03	45.29 ±3.85	39.85 ±5.63	25.49 ±3.60	24.74 ±4.97	76.61 ±3.81	24.87 ±4.17
**THP-1**	64.89 ±2.25	68.15 ±2.67	73.81 ±3.28	77.10 ±2.32	59.74 ±3.17	39.64 ±4.16	27.40 ±4.92	20.14 ±2.53	18.90 ±2.25	62.85 ±3.14	10.96 ±4.92
**K-562**	72.89 ±4.72	60.70 ±2.45	34.00 ±7.07	12.95 ±3.80	71.16 ±2.96	52.46 ±3.53	43.27 ±4.48	22.13 ±2.88	20.12 ±3.49	11.01 ±3.57	8.66 ±3.48

* Cell cycle was analyzed using PI staining and flow cytometry. The data indicate the percentage of cells in G1 phase of the cell cycle. Values represent the mean ± SD of three experiments.

### Combination treatment with DFX and DAC increased ROS levels mirroring the DAC effect

**Figure 5:**
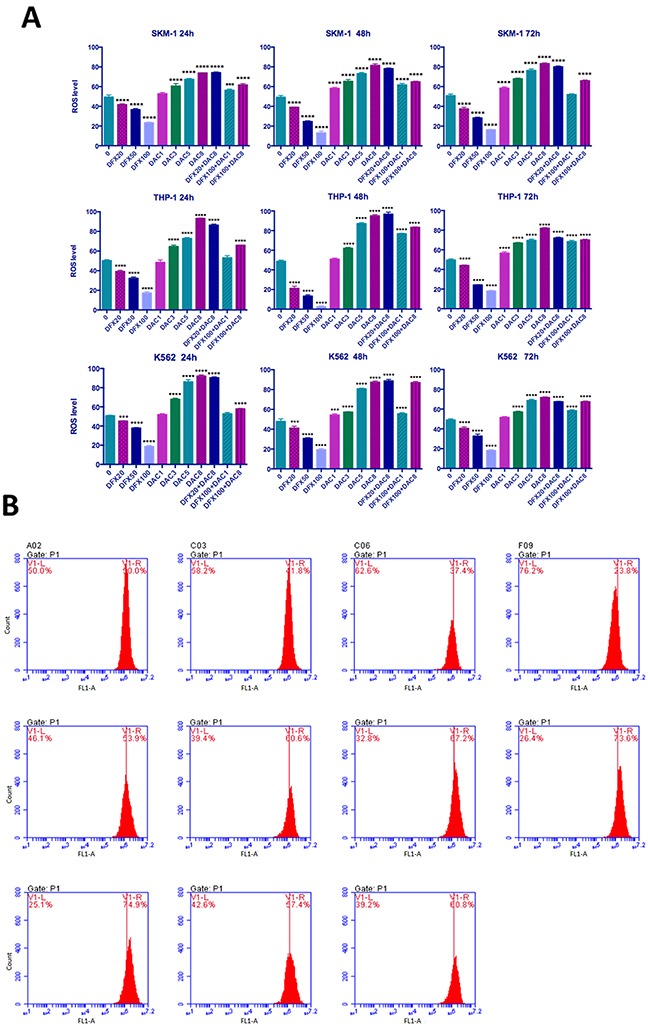
Level of ROS generation in leukemia cells **(A)** SKM-1, THP-1, and K562 cells were treated with DFX and/or DAC at different concentrations after ROS detection and then examined using flow cytometry. **(B)** SKM-1 cells treated with DFX and/or DAC at different concentrations for 72 h. ROS levels were observed to be significantly decreased with increasing DFX levels and in contrast, ROS levels increased with increasing DAC levels. The effect on ROS levels in the combination group is similar to the effect on ROS levels in the DAC group.

Since excess free iron in cells catalyzes the generation of ROS which causes oxidative stress and ROS levels can be impacted by iron chelators, we examined the ROS levels in these leukemia cell lines. We found that DFX treatment decreased cellular ROS levels in a dose-dependent manner in all three leukemia cell lines, and that DAC treatment had the opposite effect and increased ROS levels. Interestingly, the effect of the combination of the two drugs was consistent, overall trending in line with the DAC alone response. The ROS level in untreated SKM-1 cells was 49.33±2.18%. Following treatment with DFX at 20, 50, or 100 μM for 72 h, the ROS levels were 41.67±0.81%, 36.63±1.56%, and 23.37±0.59%, respectively. After treatment with DAC at 1, 3, 5, or 8 μM for 72 h, the ROS levels were 52.73±1.26%, 60.53±2.60%, 67.43±0.87%, and 73.87±0.25%, respectively. After treatment with 8 μM DAC combined with DFX at either 20 μM or 100 μM, the ROS levels were 74.20±0.70% and 61.70±1.30%, respectively. After treatment with 1 μM DAC combined with 100 μM DFX, the ROS levels was 56.37±1.17% (Figure 5A). In THP-1 and K-562 cells, the results were similar to what was seen in SKM-1 cells, as shown in Figure 5B. In conclusion, our results showed DAC has a bigger influence on ROS levels in these cell lines, compared to the effect of DFX.

### Effect of treatment with DFX and DAC alone and in combination on gene expression

**Figure 6:**
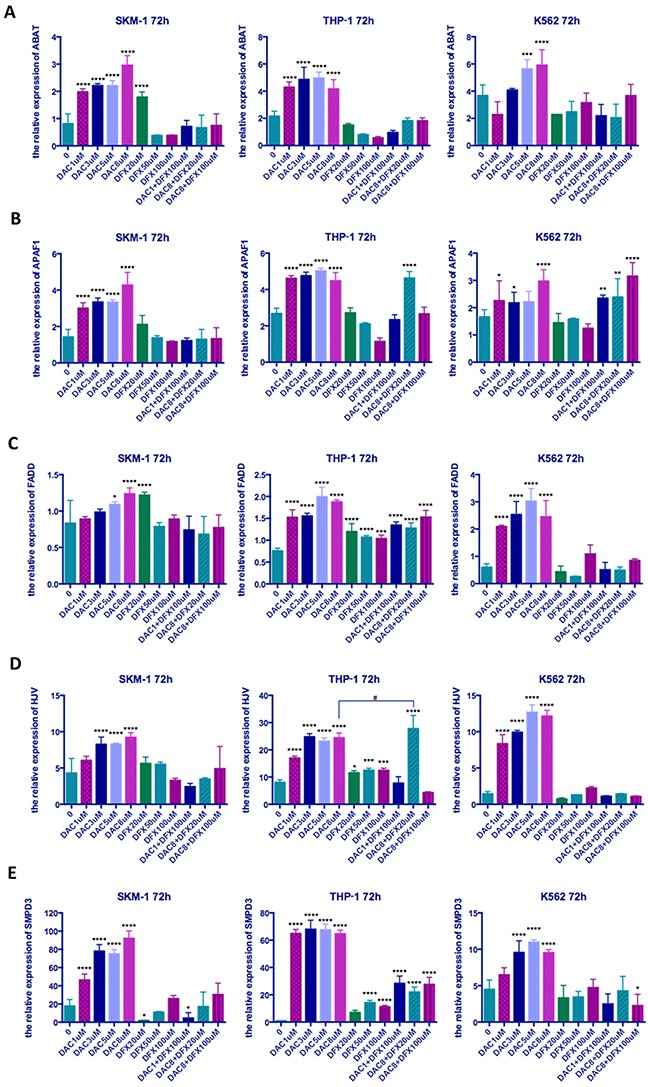
The relative expression of five genes in three leukemia cell lines following treatment at different concentrations of both drugs either alone or in combination SKM-1, THP-1, and K562 cells were treated with the indicated concentrations of DFX and/or DAC for 72 h and the expression levels of genes determined by qRT-PCR **(A)** ABAT, **(B)** APAF1, **(C)** FADD, **(D)** HJV, **(E)** SMPD. Significantly different versus control (*, P < 0.05; **, P < 0.01; ***, P < 0.001;****, P<0.0001). Significantly different between groups (#, P<0.05; ##, P<0.01; ###, P < 0.001; ####, P<0.0001).

Since DAC causes DNA demethylation, which can regulate gene expression in cis by relaxing chromatin structure [16], we analyzed the expression levels of five genes, which had been previously been shown to be hyper-methylated and down-regulated in MDS [13–15], to identify whether the comparative advantage of DAC is related to this mechanism. RT-qPCR was performed 72 h after drug treatment of cells. This time point was chosen because the highest percentage of apoptotic cells was observed in this time group. As shown in Figure 6, all five of these genes were up-regulated to varying degrees when these three leukemia cells were treated with DAC (Figure 6). However, DFX treatment alone had little impact on the expression of these genes; DFX in combination with DAC appeared to have no obvious trend. SMPD3 was the gene that showed the most sensitivity to DAC; its expression was up-regulated significantly in all cell lines and at all DAC concentrations. The magnitude of up-regulation ranged from 5.04- to 74.35-fold when these three cell lines were treated with 8 μM DAC (P<0.0001). Individual treatment with DFX up-regulated SMPD3 only in THP-1 cells at 50 and 100 μM. The combination of 1 μM DAC and 100 μM DFX up-regulated SMPD3 in SKM-1 cells (P<0.05), and it was also up-regulated at all DAC and DFX combinations in THP-1 cells (P<0.0001) (Figure 6E).

After combination treatment with 8 μM DAC and 20 μM DFX, the expression level of HJV in THP-1 cells increased significantly, which was much higher than when using 8 μM DAC alone. This study therefore demonstrated that the combination of DAC and DFX can promote re-expression of HJV.

## DISCUSSION

Most MDS patients requiring red blood cell transfusions may develop iron overload, which may adversely affect organ function and survival rate [17]. Iron chelation therapy (ICT) can effectively prevent and treat iron overload, and this has been clearly demonstrated in transfusion-dependent patients with thalassemia and MDS [18]. Recently, the French Myelodysplastic Syndromes Group (GFM) found that ICT appears to improve survival in heavily transfused lower risk MDS patients in a multicenter study and the survival advantage persisted as a powerful prognostic parameter for survival though a multivariate Cox analysis [2]. Numerous studies have shown that ICT can not only reduce the burden of iron overload as a whole, but also exist several pathogenetic mechanisms.

In our study, we investigated whether ICT has a synergistic effect with DNA methyltransferase inhibitor treatment *in vitro* in three leukemic cell lines, SKM-1, THP-1, and K-562 cells. It is worthy of note that SKM-1 is a secondary AML cell line derived from a 76-year-old Japanese male patient with MDS. SKM-1 cells show karyotype abnormalities with del(9)(q13;q22), del(17)t(17;?)(13;?), del(9q), i(17q) and t(17p), which have been detected in MDS [19]. Nakagawa *et al*. believe that this cell line may contribute to understanding the pathogenesis of MDS and its leukemic progression [20].

Deferasirox (DFX), a new oral iron chelator, was chosen as our experimental drug to treat these cell lines, either alone or in combination with the DNA methyltransferase inhibitor Decitabine (DAC). We found that both drugs, either alone or in combination, can inhibit cell growth and the level of inhibition was dose and time dependent. Both DFX [9, 10, 21] and DAC [22–24] have previously been shown to have anti-proliferative effects in K562 cells in numerous experiments, consistent with our results. We also found that these three cell lines had a greater sensitivity to DFX than DAC, with approximately an 89-94% reduction in proliferation at 100 μM DFX for 96 h and a 54-71% reduction at 8 μM DAC for 96 h compared to controls. Overall, the combination of DFX and DAC at different concentrations produced either a predominantly synergistic or additive interaction with respect to proliferation in all three cell lines.

Several studies have demonstrated that DNA methyltransferase inhibitors cause tumor cell death by inducing apoptosis. DAC has also been reported to induce apoptosis in various leukemia cell lines [11, 25, 26]. In addition DFX itself has also been shown to have strong anti-proliferative effects by inducing apoptosis in hepatocellular carcinoma cells and leukemia cell lines [9, 27, 28]. Based on these facts, we performed flow cytometry analysis using Annexin V/PI staining to determine whether the growth inhibition observed in these three cell lines, induced by the combination treatment with DFX and DAC, is related to apoptosis. The data showed that the combined use of DFX and DAC induced/caused a higher percentage of cells to undergo apoptosis compared to either DFX or DAC treatment alone (P<0.0001). Therefore, we believe these drugs inhibited the growth of leukemia cells, most likely through inducing apoptosis. We also analyzed the effect of drug treatment on the number of early apoptotic cells (annexin V+/PI-). Although the results were not as clear as for the late apoptotic cells, the trend was consistent.

However, cell cycle analysis, using DNA content, was not completely consistent in these three cell lines. DAC was shown to decrease the proportion of cells in the G0/G1 phase in all three of the leukemia cell lines, which is consistent with our previous result [29]. DFX induced G0/G1 phase cell-cycle arrest in SKM-1 and THP-1 cells but induced S phase cell-cycle arrest in K562 cells. When used in combination, the effect on the cell-cycle in SKM-1 and THP-1 cells depended on the dose of these two drugs. In K562 cells, combination treatment was additive at all concentrations except at 100 μM DFX and 8 μM DAC where it was synergistic (P<0.05). Interestingly, 8 μM DAC in combination with DFX at different concentrations, decreased the proportion of cells in the G0/G1 phase in THP-1 cells. Treatment with 8 μM DAC alone for 48 h resulted in 20.14±2.53 % of cells being in the G0/G1 phase. When combined with 20 μM or 100 μM DFX, the percentages of cells in the G0/G1 phase decreased to 18.90±2.25% and 10.96±4.92% respectively. These effects do not appear to be synergistic. Dae Sik Kim *et al*. found that treatment with DFX on its own induced an accumulation of cells in the sub-G1 phase in K562 cells through down-regulation of the NF-κB expression and β-catenin levels [21]. However, Ohyashiki *et al*. demonstrated that DFX induced cell cycle blockade in the G2-M phase by decreasing the enzyme activities of ornithine decarboxylase and spermidine N1-acetyltransferase and by decreasing ornithine decarboxylase mRNA levels [9, 30] These two published results are not consistent with each other, and neither are consistent with our data. We conclude that the effect of DFX on the cell cycle is complex and may be mediated by more than one factor, however currently we are unsure as to which factor is important.

Based on the above results, we believe that DFX does have an anti-tumor mechanism of action and showed synergistic effects with DAC in MDS and AML cell lines. Iron plays a vital role in the normal function of cells. Increasing evidence indicates the existence of an intimate link between metabolic status and epigenetic regulation in cells [12, 31]. For example, the ten-eleven translocation 1-3 (TET 1-3) proteins that catalyze the hydroxylation of 5-methylcytosine to form 5-hydroxymethylcytosine [32], are members of the superfamily of α-ketoglutarate-non-heme Fe^2+−^dependent oxygenases [33], thus providing a direct link between epigenetic regulation mechanisms and cellular iron metabolism status [12]. In cancer cells, the balance between iron metabolic status and the epigenetic regulation mechanisms is profoundly disturbed [12]. For instance, it is well established that changes in cellular iron metabolism and dysregulation of epigenetic mechanisms both play crucial roles in the progression of many types of cancer, such as breast cancer, prostate tumors, and leukemia [12, 34]. We considered the possibility of whether a similar process exists in MDS.

Numerous studies have shown that iron plays a critical role in regulating various important cellular pathways including the generation of hydroxyl radicals [35], so DFX and other chelators would be expected to lower cellular ROS levels by reducing intracellular labile iron. This has been proven to be correct in many studies and is considered to be one of the important therapeutic mechanisms for chelators [7]. In this study, DFX treatment decreased cellular ROS levels in a dose-dependent manner in all three leukemia lines, in agreement with this. In contrast, DAC treatment increased cellular ROS levels in a dose-dependent manner in all three leukemia lines. A similar result has been obtained by Fandy et al [36]. They suggested that the increase in ROS levels was deoxycytidine kinase-dependent, indicating that incorporation of DAC into nuclear DNA is required for ROS generation and that ROS accumulation in response to DAC was caspase-independent and mediated the dissipation of the mitochondrial membrane potential [36]. This effect of DAC may also show up as a comparative advantage in combination treatment; the ROS level changed in line with the individual DAC treatment in the combination group. Therefore, we conclude that the synergistic effect of DFX and DAC is not achieved by inhibiting the increase in reactive oxygen species.

Since DAC causes DNA demethylation, which can regulate gene expression in cis by relaxing chromatin structure [16], we analyzed the expression levels of five genes, which have been proven to be hyper-methylated and down-regulated in MDS [13–15], to identify whether the comparative advantage of DAC is related to this mechanism. As expected, these five genes were all up-regulated in the three leukemia cells lines when treated with DAC. In contrast, DFX treatment alone did not have an obvious effect on the expression levels of these five genes when compared with DAC treatment alone (Figure 6). The relative expression of HJV was up-regulated significantly in THP-1 cells when they were treated with 20 μM DFX in combination with 8 μM DAC and the synergistic effect was very clear (P<0.0001). The HJV gene, which encodes hemojuvelin, acts as a BMP (bone morphogenetic protein) co-receptor and triggers the binding of BMP ligands to BMP receptors to enhance hepcidin expression, which is the key factor in iron homeostasis regulation through binding to ferroportin [37, 38]. Gu *et al*. concluded that hyper-methylation of the HJV promoter region could silence gene expression and that an HJV de-methylating therapy might ameliorate iron-overload in MDS patients [15]. Although it is unknown whether the synergistic effect on HJV expression is associated with these mechanisms, we believe that through this synergistic effect, DFX may play a role in promoting the demethylation of some genes by DAC.

Our results showed that DFX and DAC both have anti-proliferative effects in these three leukemia cell lines. Combination treatment with DFX and DAC showed either an additive or a synergistic inhibition *in vitro*, which is consistent with the previous findings that iron chelation therapy can increase the survival rate of transfusion-dependent low-risk MDS patients suggested by GFM [2]. These data provide a theoretical basis for future clinical use of iron chelators and epigenetic drugs in MDS therapy and provide important information that will be useful for mechanistic studies in clinical trials.

## MATERIALS AND METHODS

### Reagents and cell culture

The iron chelator Deferasirox was kindly donated by Novartis Pharma (Alcon (China) Ophthalmic Product, Shanghai, China) and the methylation drug Decitabine was purchased from Sigma (Sigma-Alorich, Shanghai, China). Human myeloid leukemia cell lines THP-1 and K562 were obtained from the Chinese Academy of Sciences, and SKM-1 from the Japanese Collection of Research Bioresources. All cell lines used in this study were cultured in RPMI 1640 (Hyclone; GE Healthcare Life Sciences, Logan UT, USA) supplemented with 10% fetal bovine serum (FBS; Gibco; Thermo Fisher Scienti c, Inc., Waltham, MA, USA), at 37°C with 5% CO_2_.

### Drug treatment

For drug treatment, the cell lines were cultured as described above in medium supplemented with 20, 50, or 100 μmol/L DFX or 1, 3, 5, or 8 μmol/L DAC or the following combinations; 20 μmol/L DFX with 8 μmol/L DAC, 100 μmol/L DFX with 1 μmol/L DAC, or 100 μmol/L DFX with 8 μmol/L DAC for 24, 48, or 72 hours, with replacement of medium every 24 hours.

### Cell proliferation assay

Cell proliferation was measured using the Cell Counting Kit-8 assay (Dojindo Molecular Technologies, Gaithersburg, MD, USA) per the manufacturer's instructions. Briefly, aliquots (200 μL) of the cell suspension were dispensed into 96-well plates and treated with varying doses of each drug. The plates were incubated in a humidified incubator for 24, 48, 72, 96, or 120 h at 37°C with 5% CO_2_. Following this, 10 μL of CCK-8 reagent was added to each row. After incubation for 4 h, the plates were further incubated until wells with the maximum absorbance at 450 nm reached values of approximately 1 optical density (OD). Cell viability was expressed as a percentage of the control value.

### Measurement of apoptotic cells and cell-cycle distribution by flow cytometry

The leukemia cell lines SKM-1, THP-1, and K-562 (1×10^5^ cells/mL) were plated in 12-well plates, and treated with DFX and/or DAC at different concentrations for 24, 48, or 72 h. The cells were harvested, washed with phosphate-buffered saline (PBS), and then apoptosis assessed using Annexin V-FITC/Propidium Iodide (PI) staining according to the manufacturer's instructions (Dojindo Laboratories). Flow cytometry analysis was immediately performed after the incubation period. Data acquisition and analysis was performed using a BD FACS Calibur flow cytometer using FCS Express 3.0 software. Cells that were Annexin V-positive and PI-negative were considered to be the early apoptotic fraction, whereas cells that were double-positive were considered to be the late apoptotic fraction. For cell cycle analysis, cells were harvested, washed twice with ice-cold PBS, and fixed with 70% ethanol at −20°C overnight. Prior to analysis, the fixed cells were washed twice with ice-cold PBS and suspended in 1 mL of PBS containing 50 μg/mL PI for 30 min in the dark at 4°C. Cell-cycle distribution was analyzed on a BD Accuri™ C6 flow cytometer (BD, Dickinson and Company, Franklin Lakes, New Jersey, USA).

### Measurement of reactive oxygen species

Following collection, cells were incubated with DCFH-DA[2′,7′-Dichlorodihydrofluorescein diacetate] (10 μM) in the dark at 37°C for 30 min to detect ROS. The cells were washed three times with RPMI 1640 and immediately analyzed by flow cytometry. The ROS levels were analyzed with BD CFlow® software (BD, Dickinson and Company, Franklin Lakes, New Jersey, USA).

### Total RNA isolation and quantitative reverse transcription PCR

To evaluate gene expression, total RNA was extracted using TRIzol® Reagent (Invitrogen; Thermo Fisher Scientific, Inc.) according to the manufacturer's protocol. Complementary DNA (cDNA) synthesis was performed on total RNAs obtained from untreated and treated cell lines. Takara PrimeScriptTM RT Master Mix (Takara, Japan) was used for the synthesis of cDNA from cell lines as the first step for reverse transcription. For qRT-PCR, cDNA samples were amplified using the Applied BiosystemsViiA™ 7 Real-Time PCR System (Invitrogen; Thermo Fisher Scientific, Inc.) with Massimo Massimo Massimo Takara SYBR® Premix Ex TaqTM PCR reagents (Takara, Japan). The expression of ABAT, APAF-1, FADD, HJV, and SMPD3 genes was analyzed using the Applied BiosystemsViiA™ 7 software. Gene expression levels were expressed relative to the expression of GADPH.

### Statistical analysis

All data are expressed as mean ± standard deviation. Statistical comparisons between groups were performed by one-way analysis variance (ANOVA) followed by Bonferroni's multiple comparisons test. For non-parametric data, Kruskal-Wallis followed by Dunn's multiple comparison test was used. A p < 0.05 was considered statistically significant.
